# Do changes in DNA methylation mediate or interact with SNP variation? A pharmacoepigenetic analysis

**DOI:** 10.1186/s12863-018-0635-6

**Published:** 2018-09-17

**Authors:** Virginia A. Fisher, Lan Wang, Xuan Deng, Chloé Sarnowski, L. Adrienne Cupples, Ching-Ti Liu

**Affiliations:** Department of Biostatistics, Boston University School of Public Health, 801 Massachusetts Ave. 3rd floor, Boston, MA 02118 USA

**Keywords:** Causal modeling, Genomic data integration, Gene-methylation interaction, Indirect effects, Triglycerides, Genofibrate treatment

## Abstract

**Background:**

In studies with multi-omics data available, there is an opportunity to investigate interdependent mechanisms of biological causality. The GAW20 data set includes both DNA genotype and methylation measures before and after fenofibrate treatment. Using change in triglyceride (TG) levels pre- to posttreatment as outcome, we present a mediation analysis that incorporates methylation. This approach allows us to simultaneously consider a mediation hypothesis that genotype affects change in TG level by means of its effect on methylation, and an interaction hypothesis that the effect of change in methylation on change in TG levels differs by genotype. We select 322 single-nucleotide polymorphism–cytosine-phosphate-guanine (SNP-CpG) site pairs for mediation analysis on the basis of proximity and marginal genome-wide association study (GWAS) and epigenome-wide association study (EWAS) significance, and present results from the real-data sample of 407 individuals with complete genotype, methylation, TG levels, and covariate data.

**Results:**

We identified 3 SNP-CpG site pairs with significant interaction effects at a Bonferroni-corrected significance threshold of 1.55E-4. None of the analyzed sites showed significant evidence of mediation. Power analysis by simulation showed that a sample size of at least 19,500 is needed to detect nominally significant indirect effects with true effect sizes equal to the point estimates at the locus with strongest evidence of mediation.

**Conclusions:**

These results suggest that there is stronger evidence for interaction between genotype and methylation on change in triglycerides than for methylation mediating the effect of genotype.

## Background

Epigenetic mechanisms, including DNA methylation, are known to influence the phenotypic consequences of genetic variation. To fully explain the biological mechanism of an outcome of interest, it is necessary to characterize the relationship between genetic and epigenetic effects. These relationships may be described as mediation, in which genetic variation influences methylation which then influences the phenotype, or interaction (also called effect modification) in which the average effect of methylation differs by genotype, or both.

Mediation analysis has been applied to epidemiological studies of genetic and epigenetic variation to investigate the first of these hypotheses [1, 2]. Previous studies found evidence that methylation may mediate genetic risk of rheumatoid arthritis, inflammatory bowel disease, and peanut allergy [3, 4]. Gene–environment interaction methods have also been adapted to pharmacogenetics trials to address the second hypothesis.

The GAW20 data set reports a single-arm clinical trial of a drug intended to lower triglyceride (TG) levels. TG and DNA methylation are observed both before and after drug treatment. In this article, we investigate the extent to which mediation and interaction effects between single-nucleotide polymorphisms (SNPs) and changes in methylation at nearby cytosine-phosphate-guanine (CpG) sites contribute to changes in TG levels. In this context, mediation effects represent a mechanism of drug action through context-specific methylation quantitative trait loci, while interaction effects may identify genetic subgroups in which drug-induced changes in methylation lead to changes in TG levels.

## Methods

We analyzed the real GAW data set, comprising 407 individuals with complete TG, genotype, methylation, and covariate data. The sample of 679 individuals with TG, genotype, and covariate data was used for preliminary screening of SNPs for analysis. In the following, we present the details for an exposure *A* (SNP genotype alternate allele count), a continuous mediator *M* (difference in methylation posttreatment minus pretreatment), and a continuous outcome *Y* (difference in log TG posttreatment minus pretreatment). Relevant covariates *C* include age, sex, study center, and smoking status.

### Mediation hypothesis

The counterfactual approach to mediation analysis provides methods to quantify these relationships [5, 6]. This approach is based on the potential outcomes of each subject, conditional on the levels of exposure and mediator. Only one of these potential outcomes is observed for each individual, but under certain assumptions, the others may be estimated from the data. Here, *Y*_*am*_ represents the potential outcome for exposure level *A = a* and mediator level *M = m*, and *M*(*a*) represents the level of the mediator that would be observed for a given subject with exposure level *a.* The total contribution of mediation through *M* to the effect of *A* on *Y* is given by the natural indirect effect (NIE): $NIE={Y}_{aM(a)}-{Y}_{aM\left({a}^{\ast}\right)}$, which is the difference in potential outcomes among individuals with exposure level *a* compared to those with observed mediator level *M (a)* and counterfactual mediator level M (a*) which they would have had if their exposure level had been a*. For notational simplicity, we take *a = 1* and a* = 0 so the contrast is defined in terms of 1 additional alternate allele for the SNP under consideration. Note that this quantity will be zero if there is no effect of the exposure on the mediator [so that *M*(*a*) = *M*(*a*^∗^)] or no effect of the mediator on the outcome (so that ${Y}_{a{m}_1}={Y}_{a{m}_2}$for any values *m*_1_, *m*_2_ of the mediator). The NIE can be estimated from the simultaneous regression models as follows:

1 $$ E\left(M|A=a,\boldsymbol{C}=\boldsymbol{c}\right)={\beta}_0+{\beta}_1a+{\beta_2}^{\prime}\boldsymbol{c} $$

2 $$ E\left(Y|A=a,M=m,\boldsymbol{C}=\boldsymbol{c}\right)={\theta}_0+{\theta}_1a+{\theta}_2m+{\theta}_3a\ast m+{\theta}_4^{\prime}\boldsymbol{c} $$

Under the assumptions described below, the NIE=*β*_1_(*θ*_2_ + *θ*_3_). The SE of this estimate via the delta method is $\sqrt{{\Gamma \Sigma \Gamma}^{\prime }}$where Γ = (0, *θ*_2_ + *θ*_3_, 0^′^, 0, 0, *β*_1_, *β*_1_, 0^′^) and ∑ is the block-diagonal covariance matrix of the estimators from regression models (1) and (2).

This NIE estimator has a valid causal interpretation if models (1) and (2) are correctly specified and the following assumptions hold:

1. No unmeasured confounding for the exposure–outcome relationship.
2. No unmeasured confounding for the mediator–outcome relationship.
3. No unmeasured confounding for the exposure–mediator relationship.
4. No mediator-outcome confounder is affected by the exposure.

Similar assumptions are required for causal interpretation of any regression analysis.

Because the statistical power to detect indirect effects is low in studies with a small to moderate sample size, and because statistical hypothesis testing is not a valid method for qualitative assessment of confounding between the exposure and mediator, VanderWeele recommends comparing the magnitude of the total effect of the exposure on the outcome, estimated from a model that excludes the mediator, and the direct effect of exposure adjusting for the effect of the mediator and exposure–mediator interaction [6].

### Interaction hypothesis

For the purpose of assessing mediation, the interaction term in model (2) is useful primarily to allow valid estimates in the presence of non-additive contributions of the genetic and methylation effects. However, we are also interested in the interaction coefficient *θ*_3_ in its own right. The null hypothesis of interaction, *θ*_3_ = 0, may be interpreted as follows: the effect of *M* on *Y* is the same at all levels of *A*. If this null hypothesis does not hold, we may identify genotypic subgroups with different methylation effects.

### Implementation

The GAW20 real data set is drawn from a single-arm clinical trial of fenofibrate treatment in the Genetics of Lipid Lowering Drugs and Diet Network (GOLDN) study family-based cohort. We selected SNP-CpG site pairs by first running marginal association models with the phenotype:

3 $$ E\left(Y|A=a,C=c\right)={\gamma}_0+{\gamma}_1a+{\gamma_2}^{\prime }c $$

4 $$ E\left(Y|M=m,C=c\right)={\eta}_0+{\eta}_1m+{\eta_2}^{\prime }c $$

We then selected SNP-CpG site pairs with all the following 3 criteria:

1. SNP *p* value <1e-3
2. Methylation epigenome-wide association study *p* value < 0.05
3. Distance between SNP and CpG site < 50 kb pairs

These criteria were chosen to balance the considerations of low statistical power resulting from multiple testing corrections against the possibility of failing to detect significant interactions when the marginal effects are negligible.

The mediation–interaction model described above was then estimated for these SNP-CpG site pairs. The total effect refers to the coefficient *γ*_1_ in regression model (3). Models (3) and (4) were estimated genome-wide using *EPACTS*, and models (1) and (2) were estimated only at selected SNP-CpG pairs using the *kinship* and *coxme* packages in R.

Because of missing data in the posttreatment methylation data set, the sample for mediation analysis was a subset of the GWAS screening sample.

### Power calculations

We used simulation to investigate the statistical power to detect mediation between genotype and change in methylation. Based on the SNP allele frequency and distribution of change in methylation at the SNP-CpG site pair with strongest evidence of nonzero NIE, we simulated genotypes, change in methylation, and outcome measures varying the sample size, effect of SNP on change in methylation (*β*_1_), effect of methylation on outcome (*θ*_2_), and interaction effect (*θ*_3_), while holding all other model parameters constant at their observed point estimates. The simulated samples comprised unrelated individuals, so the parameters in models (1) and (2) were estimated by multiple linear regression rather than linear mixed models. All power calculations used a significance level of α = 0.05, with 500 replicates.

## Results

Using the above criteria, 322 SNP-CpG site pairs were selected, including 156 unique SNPs and 223 unique CpG sites. The maximum number of significant CpG sites within the 50-kb radius of a given SNP was 7, and the maximum number of significant SNPs within 50 kb of a given CpG site was 16. These numbers presumably reflect linkage disequilibrium (LD) patterns among nearby variants. **Tables** **1** and **2**, respectively, summarize the most significant mediation and interaction effects.

**Table 1:** Top 5 most significant NIEs

SNP	CpG	SNP MAF	Chr	Distance	TE	TE *p* value	NDE	NDE *p* value	NIE	NIE *p* value
rs12771141	cg04855826	0.359	10	12.5	0.071	3.12E − 03	1.745	0.048	0.008	0.068
rs12438405	cg21284575	0.436	15	28.8	0.064	5.29E − 03	−3.036	0.135	0.008	0.068
rs6832151	cg15003695	0.283	4	19.6	−0.074	2.77E − 03	5.253	0.008	−0.008	0.084
rs32458	cg12200124	0.329	5	39.3	0.049	0.03217	−0.272	0.398	0.007	0.088
rs11634929	cg03678138	0.041	15	8.7	−0.234	4.51E-04	−6.022	0.030	−0.054	0.089

Distance between SNP and CpG site is reported in kilobases. The natural direct effect (NDE) refers to the SNP effect that is not mediated by change in methylation. This is estimated by the coefficient ***θ***_**1**_ from model (2). The total effect (TE) is the SNP effect ***γ***_**1**_ in the unadjusted regression model (3)

*MAF* minor allele frequency

**Table 2:** Top 5 most significant interaction effects

SNP	CpG	SNP MAF	Chr	Distance	TE	TE *p* value	Int. effect	Int. *p* value	NIE	NIE *p* value
rs4686740	cg21463380	0.482	3	40.8	3.12E − 03	7.64E − 03	1.025	6.11E − 09	0.005	0.144
rs2575	cg21463380	0.482	3	47.2	5.29E − 03	9.14E − 03	0.980	3.02E − 08	0.006	0.091
rs17216446	cg15395354	0.458	4	19.0	2.77E − 03	8.50E − 04	0.624	1.41E − 06	0.003	0.257
rs1997579	cg12299303	0.118	21	39.0	0.03217	3.85E − 05	−1.339	8.95E − 04	−0.004	0.328
rs1143115	cg17140441	0.439	15	27.8	4.51E-04	3.74E − 04	0.383	1.56E − 03	0.002	0.268

Distance between SNP and CpG site in kilobases, NIE, and total genetic effects (TE) from the unadjusted model are also reported

*Int* interaction, *MAF* minor allele frequency

The Wald test for the NIE (see Table 1) reveals no SNP-CpG pairs with significant evidence of mediation at the α = 0.05 level. However, it is noteworthy that the total effect and natural direct effect show opposite direction in 3 of these 5 cases, and differ substantially in magnitude in all 5. For interaction, 3 SNP-CpG site pairs pass a Bonferroni-corrected significance threshold of 0.05/322 = 0.000155, adjusting for multiple testing at all selected pairs (see Table 2). For each of these pairs, the interaction effect was more significant than the total effect of the SNP from model (3), thereby excluding methylation. Estimated effects of methylation stratified by genotype are reported in Table 3**,** demonstrating differential responses to change in methylation. The top 2 interaction effects, both with *p* < 5e-8, were found with the same CpG site, cg21463380 on chromosome 3. Two SNPs involved in these interaction effects, rs4686740 and rs2575, are in high LD (R2 = 0.9175, D′ = 0.9675), so we assume that they are tagging the same signal. The lead SNP, rs4686740, is located in an intron of the gene *DGKG* (diacylglycerol kinase gamma), which codes an enzyme involved in lipid metabolism. The CpG site with which it interacts is located over 40 kb away, near the somatostatin coding gene *SST*. This finding suggests a regulatory relationship between this methylation site and the *DGKG* gene.

**Table 3:** Methylation effect estimates stratified by genotype at SNPs with significant interaction effects

SNP	Ref. allele	Alt. allele	No. alt alleles	No.	CpG site	Beta	SE	*p* Value
rs4686740	A	G	0	124	cg21463380	−0.11728	0.579811	0.84
			1	190		1.576491	0.558714	0.0048
			2	89		1.597208	0.594223	0.0072
rs17216446	C	A	0	124	cg15395354	0.409902	0.382098	0.28
			1	195		0.366537	0.338832	0.28
			2	76		2.026818	0.669214	0.0025

*Alt* alternate, *No* number, *Ref* reference

The second SNP-CpG pair (rs17216446- cg15395354) with significant interaction effect is located on chromosome 4 in an intron of the gene *METP1*, which codes the methionyl aminopeptidase 1 protein. The interacting CpG site is located 19 kb away, in a long noncoding RNA, BX647984. The first SNP-CpG site pair displays substantial positive methylation effect estimates for individuals with 1 or 2 G alleles, but no effect of methylation among those with homozygous reference genotype. The second pair displays a positive effect of methylation only for those with homozygous reference genotype at the SNP. It is notable that positive effects are considered deleterious in this study, as the aim of the drug treatment is to reduce TG levels. Mediation, as measured by NIE, did not reach nominal significance (*p* < 0.05) at any of the SNP-CpG sites with significant interaction effects. In all these cases, the effect of genotype on change in methylation, one factor in the product formulation of the NIE, was not significant.

**Fig. 1:**
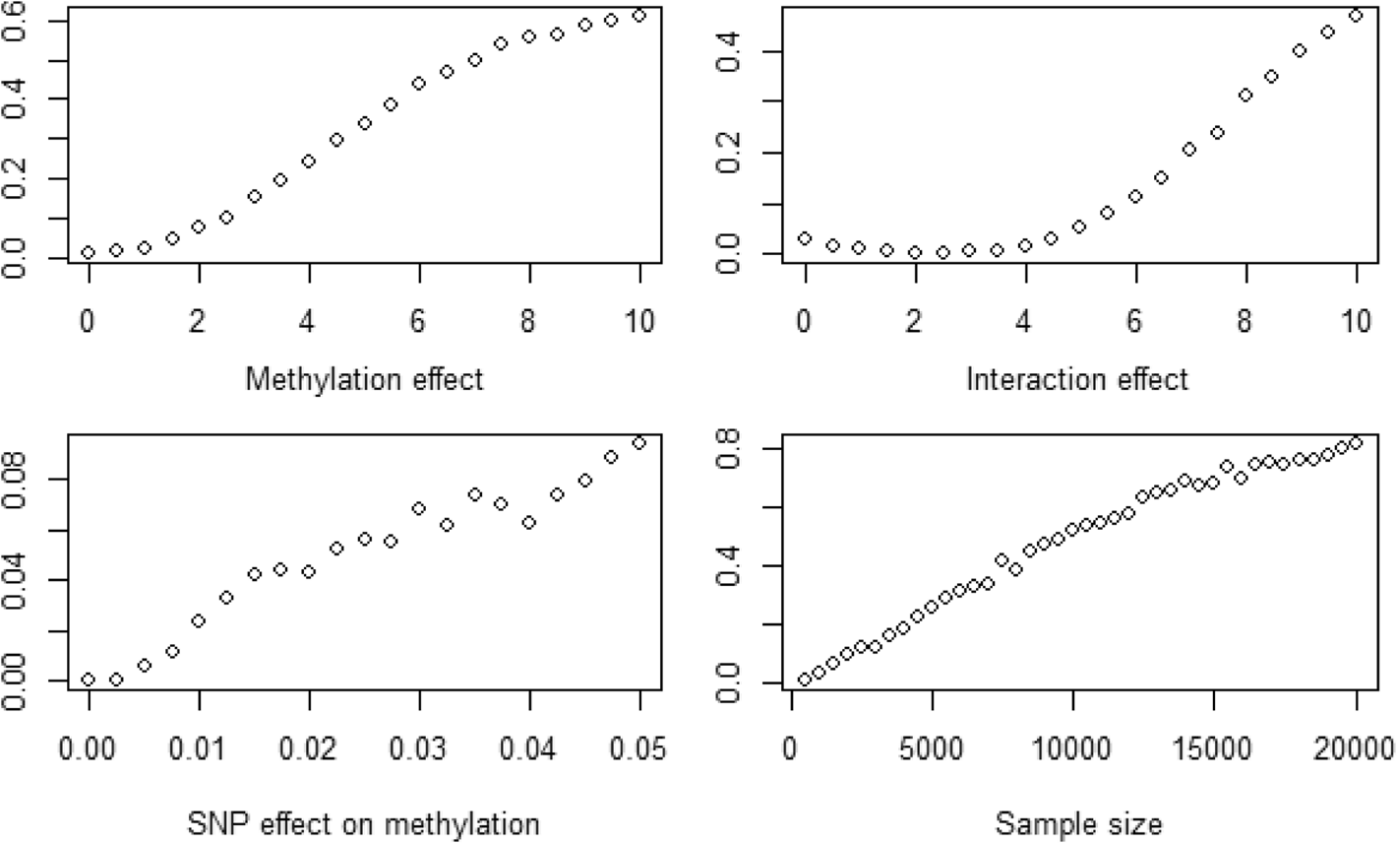
Statistical power to detect NIE as a function of (clockwise from top left) adjusted effect of methylation *θ*_2_, gene–methylation interaction effect*θ*_3_, sample size, and effect of SNP on methylation *β*_1_

Figure 1 shows plots of the statistical power from simulations to detect NIE. Varying the components of the NIE independently within the range of parameter estimates observed in the study data, all scenarios showed power of less than 50%. The genotype effect on change in methylation, *β*_1_, appears to be the greatest limitation on statistical power as increasing this parameter leads to the greatest improvements in power to detect mediation. Sample size is also a limitation, with 10,000 unrelated subjects required to attain 50% power to detect NIE, and 19,500 unrelated subjects required for 80% power, given true effect sizes of *β*_1_ = 0.001, *θ*_2_ =  − 1.661, and *θ*_3_ = 0.713, equal to the point estimates at the rs12771141-cg04855826 site.

## Discussion

The mediation analysis did not identify significant indirect effects with changes in methylation level mediating the effect of SNP genotype on change in TG levels. This may be the result of the genetic architecture of lipid traits; for example, short-term changes in DNA methylation may not be an effective mechanism for modifying TG levels. The moderately small sample size, especially in the real posttreatment methylation data, also limits our statistical power to detect indirect effects. The substantial changes in direct effect estimates after accounting for possible confounding and interaction with the nearby CpG site suggests that the effects of genotype and methylation are not independent at these sites, despite the failure to attain statistical significance. Further work is needed on hypothesis testing for mediation in the context of a heavy burden of multiple testing. In particular, statistical tests for the change in effect estimates between the unadjusted and interaction-adjusted models would provide overall quantification of the impact of methylation on genetic effects at a given locus. Furthermore, multiple-exposure or multiple-mediator models may be appropriate at loci where several SNP-CpG pairs were identified.

## Conclusions

We found significant interaction effects between SNP genotypes and CpG methylation levels on chromosomes 3 and 4. For individuals with certain genotypes, increases in methylation at the identified CpG sites were strongly associated with increased TG levels after drug treatment. These findings provide evidence of regulatory relationships between DNA methylation and SNPs at these loci. However, none of these sites showed nominally significant evidence of mediation, a consequence of a lack of association between genotype and change in methylation. In other words, the distribution of change in methylation is the same across genotypes, but the effect of change in methylation differs. This paper demonstrates the utility of integrated analysis of genetic and epigenetic data to investigate the multiple sources of variation for complex traits.

## Abbreviations

CpG: cytosine-phosphate-guanine
EWAS: epigenome-wide association study
GAW: Genetic Analysis Workshop
GWAS: genome-wide association study
LD: linkage disequilibrium
NIE: natural indirect effect
SNP: single nucleotide polymorphism
TG: triglyceride
